# Letter to the Editor regarding “Hemiarthroplasty compared to total hip arthroplasty for the treatment of femoral neck fractures: a systematic review and meta-analysis”

**DOI:** 10.1186/s13018-021-02490-7

**Published:** 2021-05-20

**Authors:** Qiujiang Li, Xingxia Long, Yinbin Wang, Xiaocheng Jiang, Lijun Cai

**Affiliations:** 1 Graduate School of Ningxia Medical University, Yinchuan, Ningxia China; 2 Department of Orthopedics, People’s Hospital of Ningxia Hui Autonomous Region, No. 56, Zhengyuan Street, Yinchuan, 750002 Ningxia China; 3 West China Hospital, Sichuan University, Chengdu, Sichuan China

Dear Editor:

We read with great interest the meta-analysis by Li et al. [1] entitled “Hemiarthroplasty compared to total hip arthroplasty for the treatment of femoral neck fractures: a systematic review and meta-analysis.” We congratulate the authors for publishing their study in this journal. Yet upon review of this article, there are serious issues that nullify the conclusions. We seek certain clarifications from the author.

Firstly, the author has extensively searched the published literature through the electronic database (Cochrane, PubMed, and Embase databases), but these databases do not seem to be sufficient to retrieve all eligible studies. Similar results have also been reported in other studies in China, while Chinese-language studies met the inclusion criteria and the author is a Chinese, so some Chinese databases (such as Wan Fang Data, CNKI databases, Vip Journal Integration Platform (VJIP), and Chinese BioMedical databases) should also be included, which may contribute to a more comprehensive collection of qualified studies.

Secondly, it is general that RCTs and observational studies cannot be combined unless the results are related to the harmful/adverse effects of the intervention as described in the Cochrane Handbook for Systematic Reviews [2]. Therefore, it is a methodological error to combine RCTs with cohort studies in the meta-analysis. A better solution is to make a subgroup analysis of RCTs and cohort studies.

Thirdly, only two RCT studies were included in this meta-analysis, which makes it difficult to ensure data of relatively high quality. A more serious problem is that it was not possible to include all articles published before August 2019, especially some high-quality RCT studies. As far as we know, five RCTs [3–7] and one CCT [8] studies were published before the deadline but were not included. As a supplement, we would like to provide more information about these six articles. The studies were eligible according to the authors’ inclusion criteria, and they are very helpful in making strong conclusions. Details of these six studies are shown in Table 1.

**Table 1:** Six studies not included in the Li et al. [1] systematic review

Study	Year	No. of patients		Age (years)		Gender		Mean follow-up duration (months)	Study design
		HA	TKA	HA	TKA	HA	TKA		
Tol et al. [3] and van den Bekerom et al. [5]	2017 and 2010	137	115	80.3	82.1	115F	90F	144	RCT
Avery et al. [4] and Baker et al. [7]	2011 and 2006	41	40	75.8	74.2	NA	NA	108	RCT
Keating et al. [6]	2006	69	69	75.0	75.2	54F	52F	24	RCT
Mouzopoulos et al. [8]	2008	34	37	74.2	73.1	24F	28F	60	CCT

*RCT* randomized controlled trial, *CCT* controlled clinical trial, *NA* not available, *HA* hemiarthroplasty, *TKA* total hip arthroplasty

Fourthly, it was obvious that the two literatures [9, 10] are published by the same lead author. The authors repeatedly extracted data from both literatures for analysis, which could lead to erroneous conclusions and mislead clinical practice. Therefore, if several articles were published in the same trial, the study with the most relevant information or the longest follow-up period may be the most appropriate.

Fifthly, the full results of the meta-analysis show a high degree of heterogeneity and sensitivity analysis was lacking to further analyze the sources of heterogeneity; thus, it weakens the credibility of the results.

Finally, all of the above points may lead readers to question the reliability of the conclusions. Therefore, we hope that the authors will correct the relevant problems pointed out, which will only serve to benefit the research community at large.

## Data Availability

Not applicable.
